# Ferroptosis in Differentiated Thyroid Cancer: Redox–Iodine Metabolism, Dedifferentiation, and Therapeutic Sensitization Beyond Anaplastic Disease

**DOI:** 10.3390/cells15070630

**Published:** 2026-03-31

**Authors:** Jaewang Lee, Jong-Lyel Roh

**Affiliations:** 1 Department of Otorhinolaryngology-Head and Neck Surgery, CHA Bundang Medical Center, CHA University, Seongnam 13496, Republic of Korea; 2 Logsynk, Seoul 06164, Republic of Korea; 3 Department of Biomedical Science, General Graduate School, CHA University, Pocheon 11160, Republic of Korea

**Keywords:** differentiated thyroid cancer, ferroptosis, radioiodine-refractory, redox metabolism, therapy resistance

## Abstract

**Highlights:**

**What are the main findings?**

- Ferroptosis in differentiated thyroid cancer (DTC) is shaped by thyroid-specific redox and iodine metabolism.
- Dedifferentiation and radioiodine refractoriness are closely linked to ferroptosis resistance mechanisms.
- Lipid metabolic remodeling and antioxidant adaptation dynamically regulate ferroptosis susceptibility in DTC.

**What are the implications of the main findings?**

- Ferroptosis represents a context-dependent vulnerability rather than a universal cell death pathway in DTC.
- Modulating ferroptosis may sensitize radioiodine-refractory tumors to existing therapeutic strategies.
- Integrating ferroptosis biology into treatment design offers new opportunities to overcome therapy resistance.

**Abstract:**

Differentiated thyroid cancer (DTC), including papillary and follicular subtypes, is generally associated with favorable prognosis; however, a subset of patients develops recurrent, metastatic, or radioiodine-refractory diseases with limited therapeutic options. Ferroptosis, an iron-dependent form of regulated cell death driven by lipid peroxidation, has recently emerged as a biologically relevant process in thyroid cancer, yet its role in differentiated disease remains incompletely defined. Unlike many other malignancies, thyroid cancer arises within an organ intrinsically shaped by iodine-dependent redox reactions required for thyroid hormone biosynthesis. This unique oxidative environment imposes selective pressure on tumor cells to adapt redox balance, lipid metabolism, and antioxidant defenses, all of which are central regulators of ferroptosis. Accumulating evidence indicates that ferroptosis susceptibility in DTC is dynamically modulated by differentiation status, oncogenic signaling, metabolic rewiring, and tumor microenvironmental interactions. Notably, progression toward radioiodine-refractory disease is accompanied by dedifferentiation and reinforcement of anti-ferroptotic programs, linking ferroptosis resistance to therapeutic failure. In this review, we synthesize recent original studies and contemporary reviews to provide a focused overview of ferroptosis in DTC, excluding anaplastic disease. We discuss thyroid-specific redox and iodine metabolism, genetic and metabolic determinants of ferroptosis sensitivity, lipid remodeling, and immune–microenvironmental interactions, and highlight translational opportunities for targeting ferroptosis in radioiodine-refractory DTC. By reframing ferroptosis as a context-dependent vulnerability rather than a universal death pathway, this review outlines a conceptual roadmap for integrating ferroptosis modulation into existing therapeutic strategies for DTC.

## 1. Introduction

Differentiated thyroid cancer (DTC), including papillary and follicular thyroid carcinoma, accounts for the majority of thyroid malignancies and is generally associated with favorable long-term survival [1]. Nevertheless, a clinically meaningful proportion of patients develop recurrent, metastatic, or radioiodine-refractory disease, which remains the principal cause of thyroid-cancer-related mortality [2]. Once resistance to radioactive iodine (RAI) therapy occurs, therapeutic options become limited. Disease control increasingly relies on targeted systemic therapies, which often show variable and transient efficacy [3,4]. These clinical challenges have stimulated interest in alternative forms of regulated cell death that may represent exploitable vulnerabilities in thyroid cancer. Ferroptosis, an iron-dependent form of regulated cell death driven by lipid peroxidation, has emerged as a biologically and therapeutically relevant process across multiple malignancies [5,6]. Unlike apoptosis, ferroptosis is tightly linked to cellular metabolism, redox balance, and membrane lipid composition, positioning it at the intersection of tumor biology and therapeutic stress responses [7,8].

The thyroid gland provides a distinctive biological context for ferroptosis research. Thyroid follicular cells are intrinsically exposed to sustained oxidative reactions required for thyroid hormone biosynthesis, rendering redox homeostasis a central feature of both normal thyroid physiology and thyroid tumorigenesis [9]. Malignant transformation and progression, therefore, occur within a pre-existing oxidative environment, suggesting that thyroid cancers may rely on unique adaptive mechanisms to regulate oxidative stress and cell survival [10]. Over the past several years, increasing evidence has linked ferroptosis-related pathways to thyroid cancer biology. Original studies and bioinformatic analyses have reported associations between ferroptosis-related gene signatures and tumor progression, prognosis, immune infiltration, and therapeutic response in thyroid cancer cohorts [11,12,13]. Experimental investigations have further demonstrated that modulation of key ferroptosis regulators can influence thyroid cancer cell viability and sensitivity to anticancer therapies [14,15]. Collectively, these findings have established ferroptosis as a relevant biological process in thyroid cancer.

However, most existing reviews have approached ferroptosis in thyroid cancer from a broad or pan-cancer perspective, frequently emphasizing anaplastic thyroid cancer because of its aggressive phenotype and experimental tractability [16,17,18,19]. While these reviews have provided valuable mechanistic overviews, they often underrepresent the biological and clinical questions specific to DTC. Importantly, prior thyroid-cancer-focused reviews have largely remained descriptive, with limited integration of thyroid-specific physiology—particularly iodine-dependent redox metabolism—or the dynamic relationship between ferroptosis and tumor differentiation status. In addition, the potential link between ferroptosis regulation and the development of radioiodine refractoriness has not been systematically conceptualized. DTC differs fundamentally from anaplastic disease in differentiation status, iodine metabolism, oncogenic drivers, and treatment paradigms, underscoring the need for a focused reassessment [20]. In this context, the present review differs from prior work by providing a DTC-centered framework that integrates thyroid-specific redox–iodine biology, dedifferentiation-associated metabolic reprogramming, and ferroptosis susceptibility as a dynamically regulated process rather than a static cell death mechanism. In DTC, ferroptosis should be considered not merely as an alternative mode of cell death but as a process intricately linked to tumor differentiation, metabolic adaptation, and therapeutic resistance [21,22]. The development of radioiodine refractoriness and dedifferentiation is accompanied by extensive redox and metabolic reprogramming, processes that overlap with established ferroptosis resistance mechanisms [23]. These observations raise critical questions regarding how ferroptosis susceptibility evolves during DTC progression and how it may be therapeutically manipulated [24].

Therefore, a comprehensive and updated synthesis focused specifically on DTC is warranted. This review aims to integrate recent original studies and contemporary reviews to examine ferroptosis within the unique redox and metabolic context of DTC, with particular attention to differentiation status, tumor microenvironment, and translational implications for radioiodine-refractory disease. By reframing ferroptosis as a context-dependent vulnerability rather than a universal death pathway, this review seeks to clarify its biological significance and therapeutic potential in DTC.

## 2. Core Ferroptosis Machinery: General Mechanisms Relevant to Cancer

**Figure 1:**
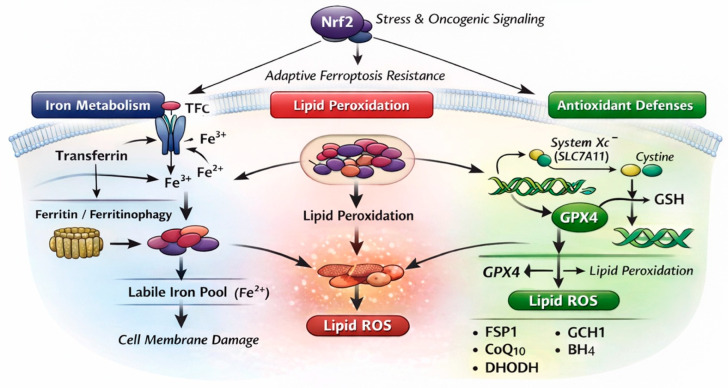
**Core ferroptosis machinery relevant to differentiated thyroid cancer.** Ferroptosis is a regulated form of cell death governed by three interdependent axes: iron metabolism, lipid peroxidation, and antioxidant defense systems. Iron uptake through transferrin–transferrin receptor signaling and ferritinophagy contributes to the labile iron pool, which catalyzes lipid peroxidation via iron-dependent reactions. Polyunsaturated-fatty-acid-containing phospholipids serve as substrates for lipid peroxidation, leading to membrane damage and ferroptotic cell death. Antioxidant defense pathways, centered on the cystine–glutathione (GSH)–GPX4 axis, suppress lipid peroxidation and limit ferroptosis. GPX4-independent mechanisms, including the FSP1–CoQ, GCH1–BH_4_, and DHODH pathways, provide additional layers of protection. In differentiated thyroid cancer (DTC), chronic redox stress and oncogenic signaling activate Nrf2-dependent adaptive programs that reinforce ferroptosis resistance and shape context-dependent susceptibility. These pathways are functionally interconnected and collectively determine ferroptosis susceptibility rather than acting as independent modules. Abbreviations: CoQ_10_, coenzyme Q_10_; DHODH, dihydroorotate dehydrogenase; Fe^3+^, ferric iron; Fe^2+^, ferrous iron; FSP1, ferroptosis suppressor protein 1; GCH1, GTP cyclohydrolase 1; GPX4, glutathione peroxidase 4; GSH, glutathione; Lipid ROS, lipid reactive oxygen species; Nrf2, nuclear-factor-erythroid-2-related factor 2; System Xc^−^ (SLC7A11), cystine/glutamate antiporter; TfR, transferrin receptor.

Ferroptosis is a regulated form of cell death driven by iron-dependent lipid peroxidation and the failure of cellular antioxidant systems, fundamentally distinct from apoptosis, necroptosis, and other non-apoptotic death programs [5,6]. Since its initial characterization, ferroptosis has been increasingly recognized as a context-dependent vulnerability in cancer cells, shaped by metabolic state, redox balance, and microenvironmental cues [25,26]. Emerging evidence suggests that canonical ferroptosis machinery intersects with organ-specific redox biology and differentiation status, necessitating a tailored conceptual framework [15]. At its core, ferroptosis is governed by three interdependent axes: iron metabolism, lipid peroxidation, and antioxidant defense systems [27] (Figure 1). Dysregulation of any one of these axes can tip the balance toward ferroptotic cell death, while adaptive remodeling across these pathways confers ferroptosis resistance [28]. Thyroid cancer cells, particularly those undergoing dedifferentiation or therapeutic pressure, appear to exploit this plasticity to survive under sustained oxidative stress [29,30]. This section summarizes the general molecular framework of ferroptosis that is broadly applicable across cancer types. Thyroid-specific features that modulate these pathways are discussed separately in the following section.

### 2.1. Iron Metabolism and Labile Iron Pools

Iron is an essential cofactor for ferroptosis, as redox-active ferrous iron (Fe^2+^) catalyzes the formation of lipid radicals through Fenton chemistry and iron-dependent enzymatic reactions [31]. Cellular iron homeostasis is regulated by iron uptake (transferrin receptor 1, TFRC), storage (ferritin), export (ferroportin), and autophagic ferritin degradation (ferritinophagy), collectively determining the size of the labile iron pool [32]. Alterations in iron metabolism have been increasingly reported. Transcriptomic analyses have identified differential expression of iron-handling genes correlating with prognosis and ferroptosis-related gene signatures in DTC cohorts [33,34]. Experimental studies suggest that the modulation of ferritinophagy and intracellular iron availability can influence thyroid cancer cell sensitivity to ferroptosis inducers, although findings remain heterogeneous across models [35]. Importantly, the thyroid gland’s intrinsic exposure to oxidative reactions during hormone synthesis may impose selective pressure favoring tight iron sequestration, thereby limiting ferroptotic vulnerability in malignant cells [21]. However, these findings remain inconsistent across experimental systems, with some studies reporting increased iron availability promoting ferroptosis sensitivity, whereas others suggest that enhanced iron sequestration predominates in thyroid cancer cells. This discrepancy likely reflects differences in tumor models, differentiation status, and redox context, and underscores the need for cautious interpretation when extrapolating these findings to DTC.

### 2.2. Lipid Peroxidation and Membrane Susceptibility

Lipid peroxidation of polyunsaturated fatty acid (PUFA)-containing phospholipids represents the terminal step of ferroptosis and determines membrane vulnerability to oxidative damage [7,36]. The incorporation of PUFAs into membrane phospholipids is regulated by acyl-CoA synthetase long-chain family member 4 (ACSL4) and lysophosphatidylcholine acyltransferase 3 (LPCAT3), which together dictate membrane susceptibility to oxidative damage [37,38]. Several studies have implicated lipid metabolic remodeling in thyroid cancer progression and ferroptosis resistance. Reduced ACSL4 expression, increased monounsaturated fatty acid (MUFA) content, and enhanced lipid droplet formation have been proposed as adaptive mechanisms that buffer lipid peroxidation stress in thyroid cancer cells [39,40]. These adaptations are particularly relevant in DTC, where long-term survival under chronic oxidative pressure may select for membrane compositions less prone to ferroptotic damage. Such lipid remodeling is increasingly recognized as a hallmark of dedifferentiation and therapy resistance across cancers, including thyroid malignancies [18,41].

### 2.3. Antioxidant Defense Systems: GPX4-Centered and GPX4-Independent Pathways

The glutathione peroxidase 4 (GPX4)–glutathione (GSH) axis represents the central antioxidant system suppressing ferroptosis by detoxifying lipid hydroperoxides [42]. System Xc^−^, composed of SLC7A11 and SLC3A2, maintains intracellular cysteine availability for GSH synthesis and thereby indirectly sustains GPX4 activity. Upregulation of GPX4 and SLC7A11 has been consistently associated with enhanced cell survival, aggressive features, and resistance to oxidative stress-inducing therapies [43,44,45,46]. These findings align with the notion that thyroid cancer cells rely heavily on antioxidant buffering to counterbalance both intrinsic and therapy-induced oxidative stress. Moreover, recent studies suggest that GPX4 expression may increase during progression to radioactive iodine-refractory (RAIR) DTC, linking ferroptosis resistance to loss of differentiation and RAI refractoriness [44,47]. Beyond GPX4, several GPX4-independent ferroptosis defense pathways have been identified, including ferroptosis suppressor protein 1 (FSP1)-mediated ubiquinol regeneration, the GTP cyclohydrolase 1 (GCH1)–tetrahydrobiopterin (BH_4_) axis, and mitochondrial dihydroorotate dehydrogenase (DHODH)-dependent CoQH_2_ production [48,49,50]. While direct functional evidence for these GPX4-independent pathways in DTC remains limited, most current insights are derived from pan-cancer analyses or transcriptomic datasets rather than thyroid-specific experimental validation [12,51]. Therefore, their relative contribution to ferroptosis resistance in DTC should be interpreted with caution.

### 2.4. Redox Signaling and Adaptive Ferroptosis Resistance

Redox-regulatory transcription factors, most notably nuclear-factor-erythroid-2-related factor 2 (Nrf2), play a pivotal role in coordinating antioxidant responses and ferroptosis resistance [52,53]. Activation of Nrf2 signaling induces expression of SLC7A11, GPX4, ferritin, and NADPH-generating enzymes, collectively reinforcing ferroptosis defense [54,55]. In DTC, aberrant activation of Nrf2 has been linked to tumor progression, metabolic reprogramming, and resistance to targeted therapies [56,57,58]. Given the thyroid gland’s dependence on finely tuned redox control, Nrf2-mediated adaptation may represent a particularly potent barrier to ferroptosis induction in DTC, especially under therapeutic stress.

### 2.5. Conceptual Integration for Thyroid Cancer

Taken together, the ferroptosis core machinery in thyroid cancer can be viewed as a dynamically regulated network shaped by iron availability, membrane lipid composition, and layered antioxidant defenses. Unlike rapidly proliferating, highly unstable tumors such as anaplastic thyroid cancer, DTC appears to evolve ferroptosis resistance gradually, in parallel with dedifferentiation, redox adaptation, and therapeutic selection pressure. This adaptive trajectory underscores the importance of contextualizing ferroptosis not as a binary on–off switch but as a tunable vulnerability influenced by thyroid-specific biology. Importantly, these ferroptosis-regulatory axes do not operate independently but are tightly interconnected within a coordinated network. Nrf2 activation not only enhances antioxidant capacity through SLC7A11 and GPX4 induction but also promotes NADPH generation and lipid metabolic reprogramming, thereby limiting the accumulation of peroxidizable phospholipids. Concurrently, lipid remodeling—through reduced PUFA incorporation, increased MUFA synthesis, and lipid droplet formation—decreases substrate availability for lipid peroxidation, functionally complementing antioxidant defenses. Thus, ferroptosis resistance in DTC emerges from the convergence of redox signaling, metabolic adaptation, and membrane remodeling, rather than from alterations in a single pathway. This integrated network is further shaped by thyroid-specific oxidative biology and evolves dynamically during dedifferentiation and therapeutic stress.

## 3. Thyroid-Specific Redox and Iodine Metabolism as a Ferroptosis-Modulating Context

**Figure 2:**
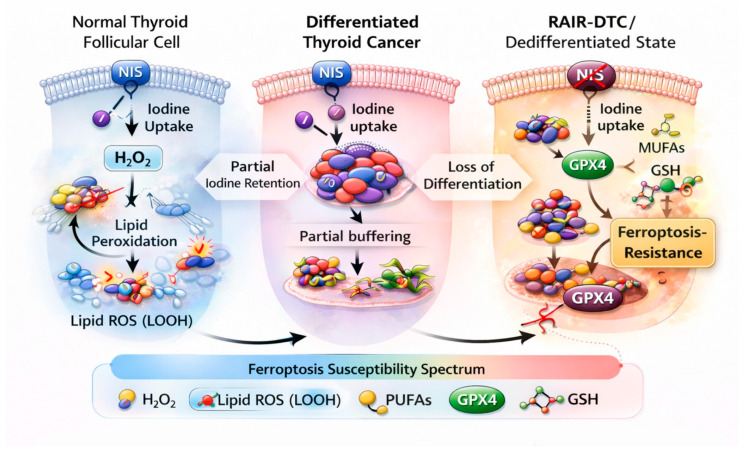
**Thyroid-specific redox biology and lipid remodeling dynamically regulate ferroptosis susceptibility during dedifferentiation.** The thyroid gland is characterized by intrinsic oxidative stress driven by iodine oxidation and hydrogen peroxide (H_2_O_2_) production required for thyroid hormone biosynthesis. In normal thyroid follicular cells, iodine uptake via the sodium–iodide symporter (NIS) and tightly regulated redox homeostasis maintain a balance between lipid peroxidation and antioxidant defenses. In DTC, partial retention of iodine metabolism is accompanied by adaptive buffering of oxidative stress, resulting in context-dependent ferroptosis susceptibility. Progressive dedifferentiation and the development of radioiodine-refractory disease are associated with loss of NIS expression, lipid metabolic remodeling favoring monounsaturated fatty acids and lipid droplet accumulation, and reinforcement of antioxidant defense programs such as GPX4 upregulation. These coordinated changes limit lipid peroxidation and promote ferroptosis resistance, highlighting ferroptosis susceptibility as a dynamic, differentiation-state-dependent property in thyroid cancer. These features highlight how thyroid-specific redox biology modifies the general ferroptosis framework. Abbreviations: DTC, differentiated thyroid cancer; GSH, glutathione; GPX4, glutathione peroxidase 4; H_2_O_2_, hydrogen peroxide; LOOH, lipid hydroperoxides; MUFAs, monounsaturated fatty acids; NIS, sodium/iodide symporter; PUFAs, polyunsaturated fatty acids; RAIR-DTC, radioiodine-refractory differentiated thyroid cancer; ROS, reactive oxygen species.

In contrast to the general ferroptosis machinery described above, the thyroid gland provides a unique physiological context in which ferroptosis regulation is shaped by iodine-dependent redox metabolism and sustained oxidative stress. The thyroid gland is unique among human organs in that its physiological function intrinsically depends on sustained oxidative reactions. Thyroid hormone biosynthesis requires continuous production of hydrogen peroxide (H_2_O_2_) at the apical membrane of follicular cells to catalyze iodide oxidation and iodination of thyroglobulin, rendering the thyroid one of the most oxidatively active tissues under normal conditions [59]. This distinctive redox environment imposes a chronic oxidative burden on thyroid follicular cells, necessitating tightly regulated antioxidant systems to maintain cellular integrity [9]. Malignant transformation of thyroid follicular cells, therefore, occurs within a pre-existing oxidative milieu, fundamentally distinguishing thyroid cancer from many other epithelial malignancies [60]. Accumulating evidence suggests that redox adaptation is not merely a bystander phenomenon but a central driver of thyroid tumor initiation, progression, and therapeutic response [61,62] (Figure 2). In this context, ferroptosis—defined by iron-dependent lipid peroxidation exceeding antioxidant buffering capacity—represents a particularly relevant cell death modality whose regulation is likely shaped by thyroid-specific biology (Table 1).

### 3.1. Physiological Oxidative Stress and Ferroptosis Susceptibility

Physiological H_2_O_2_ generation in thyroid follicular cells is mediated primarily by dual oxidases (DUOX1 and DUOX2), which localize to the apical membrane and generate reactive oxygen species (ROS) in a spatially controlled manner [63]. While this system is tightly regulated in normal thyroid tissue, dysregulation of DUOX activity and antioxidant defenses has been implicated in thyroid carcinogenesis [64]. Persistent oxidative stress may promote genomic instability, lipid peroxidation, and metabolic reprogramming, creating selective pressure for cancer cells capable of surviving under redox imbalance [65]. From a ferroptosis perspective, this environment presents a paradox. This creates a paradox in which oxidative stress both promotes lipid peroxidation and selects for robust anti-ferroptotic adaptations. In DTC, this balance appears to shift toward progressive reinforcement of ferroptosis resistance [24]. DTC appears to resolve this paradox by progressively strengthening anti-ferroptotic defenses while maintaining sufficient redox flexibility to support survival and proliferation. Notably, the dual role of oxidative stress in thyroid cancer remains unresolved. While chronic oxidative exposure may predispose cells to lipid peroxidation and ferroptosis, it simultaneously selects for robust antioxidant adaptations, potentially overriding ferroptotic vulnerability. This paradox has not been systematically addressed in DTC and represents a key area of uncertainty.

### 3.2. Iodine Metabolism, Differentiation Status, and Redox Remodeling

Iodine uptake and organification are hallmarks of differentiated thyroid follicular cells and are critically dependent on the sodium–iodide symporter (NIS) and associated iodide-handling machinery [66]. Loss of iodine avidity, a defining feature of RAIR-DTC, reflects a broader process of dedifferentiation accompanied by profound metabolic and transcriptional reprogramming [67]. Several studies have suggested that dedifferentiation is associated with attenuation of thyroid-specific oxidative processes, including reduced iodide transport and altered H_2_O_2_ dynamics [68]. This shift may paradoxically decrease overt oxidative stress while simultaneously activating compensatory antioxidant and survival pathways. Such adaptations overlap substantially with known ferroptosis resistance mechanisms, including upregulation of GPX4, SLC7A11, ferritin, and NADPH-generating enzymes [69,70,71]. Importantly, this redox remodeling is not binary but exists along a continuum, mirroring the gradual loss of differentiation observed clinically in DTC progression. As tumors transition from iodine-avid to iodine-refractory states, ferroptosis susceptibility may be dynamically reshaped rather than uniformly suppressed, suggesting potential therapeutic windows during which ferroptosis induction could be exploited.

### 3.3. Nrf2-Centered Antioxidant Programs in Thyroid Cancer

The Nrf2 pathway plays a pivotal role in orchestrating antioxidant responses and metabolic adaptation under oxidative stress [72]. Aberrant activation of Nrf2 signaling has been reported across multiple studies and is associated with tumor aggressiveness, metabolic rewiring, and resistance to therapy [73]. Nrf2 activation induces a coordinated transcriptional program encompassing cystine uptake (SLC7A11), GSH synthesis, GPX4 expression, iron storage, and lipid metabolism, collectively reinforcing ferroptosis resistance [74]. Given the thyroid gland’s reliance on redox homeostasis, Nrf2-mediated adaptation may be particularly advantageous for malignant thyroid cells, enabling survival under both intrinsic oxidative stress and therapy-induced redox perturbations [56,57]. Notably, recent bioinformatic analyses of DTC cohorts have identified ferroptosis-related gene signatures enriched for Nrf2 target genes, correlating with unfavorable prognosis and immune exclusion phenotypes [58,75]. These findings underscore the clinical relevance of redox-driven ferroptosis modulation in DTC.

### 3.4. Iron Handling in an Oxidatively Active Organ

Iron metabolism intersects with thyroid redox biology at multiple levels. Iron is required for thyroid peroxidase activity and normal hormone synthesis, yet excess labile iron can exacerbate oxidative damage through Fenton chemistry [76]. Thus, tight regulation of iron uptake, storage, and utilization is essential in thyroid tissue. Dysregulated iron handling has been implicated in ferroptosis resistance and tumor progression, although reported findings are heterogeneous [18,77]. Increased ferritin expression and altered ferritinophagy have been proposed as mechanisms to limit labile iron availability, thereby constraining lipid peroxidation despite an oxidative environment [35]. Such adaptations may be particularly pronounced in differentiated tumors that must balance iron-dependent physiological functions with protection against ferroptotic death.

### 3.5. Implications for Ferroptosis in Differentiated Thyroid Cancer

Collectively, the thyroid-specific redox and iodine metabolic context provides a compelling framework for understanding ferroptosis regulation in DTC. Unlike cancers that acquire oxidative stress primarily as a consequence of malignant metabolism, thyroid cancers originate and evolve within an organ intrinsically shaped by redox chemistry. This background fundamentally influences how ferroptosis is sensed, resisted, or exploited during tumor progression. The progressive redox remodeling accompanying dedifferentiation and RAI refractoriness suggests that ferroptosis susceptibility is not static but dynamically regulated in DTC. This insight has important translational implications. Therapeutic strategies aimed at inducing ferroptosis may need to account for differentiation status, iodine metabolism, and antioxidant capacity to achieve meaningful efficacy. Conversely, redifferentiation therapies that restore iodine handling may inadvertently reshape ferroptosis sensitivity, offering opportunities for rational combination strategies [78]. In summary, ferroptosis in DTC must be interpreted through the lens of thyroid-specific redox and iodine metabolism. Recognizing this organ-specific context not only clarifies inconsistencies across experimental studies but also provides a conceptual foundation for integrating ferroptosis-targeted approaches into existing therapeutic paradigms for DTC and RAIR-DTC. Importantly, thyroid-specific redox and iodine metabolism should not be viewed as upstream regulators alone but as integral components of the ferroptosis-regulatory network. Chronic oxidative exposure influences not only antioxidant defenses but also lipid remodeling and iron handling, thereby simultaneously modulating multiple ferroptosis-controlling axes. Thus, while the core ferroptosis machinery remains conserved, thyroid-specific redox and iodine metabolism fundamentally reshape how these pathways are engaged, distinguishing DTC from other cancer types.

## 4. Genetic, Epigenetic, and Metabolic Determinants of Ferroptosis Sensitivity in Differentiated Thyroid Cancer

Ferroptosis sensitivity in cancer cells is increasingly recognized as a genetically and metabolically programmed trait rather than a uniform cellular response to oxidative stress [22]. In DTC, tumor behavior is strongly shaped by specific oncogenic drivers, differentiation status, and metabolic adaptations, all of which intersect with ferroptosis-regulatory networks. Understanding how these determinants modulate ferroptosis susceptibility is critical for translating mechanistic insights into clinically meaningful strategies.

### 4.1. Oncogenic Drivers and Ferroptosis Regulation in DTC

Papillary and follicular thyroid carcinomas are characterized by recurrent genetic alterations involving the MAPK and PI3K signaling pathways, including BRAF^V600E^, RAS mutations, RET/PTC rearrangements, and PAX8–PPARG fusions [79,80]. These oncogenic drivers not only dictate tumor initiation and progression but also influence cellular metabolism, redox balance, and differentiation state, thereby indirectly shaping ferroptosis sensitivity. BRAF^V600E^-positive thyroid cancers exhibit pronounced MAPK pathway activation, which is closely linked to dedifferentiation, loss of thyroid-specific gene expression, and resistance to radioactive iodine therapy [69,81]. MAPK hyperactivation has been shown to induce antioxidant and cytoprotective programs, including upregulation of Nrf2 signaling and GSH metabolism, which are key components of ferroptosis resistance [82,83].

Consistent with this, bioinformatic analyses of DTC cohorts have demonstrated that tumors harboring BRAF mutations often exhibit ferroptosis-related gene expression profiles indicative of reduced ferroptotic susceptibility [84]. RAS-driven thyroid cancers, while often retaining a more differentiated phenotype compared with BRAF-mutant tumors, also display metabolic rewiring that may influence ferroptosis sensitivity [81]. RAS signaling has been linked to altered lipid metabolism and increased reliance on antioxidant buffering, suggesting context-dependent effects on ferroptotic vulnerability [23]. These observations highlight that oncogenic signaling pathways in DTC do not uniformly suppress or promote ferroptosis but instead modulate ferroptosis-related pathways in driver-specific manners.

### 4.2. Dedifferentiation as a Ferroptosis-Resistant State

Dedifferentiation represents a pivotal biological transition in DTC progression, marked by loss of thyroid-specific functions, reduced iodine uptake, and acquisition of more aggressive phenotypes [20]. This process is accompanied by extensive transcriptional and metabolic reprogramming that parallels known ferroptosis resistance mechanisms. Multiple studies have shown that dedifferentiated thyroid cancer cells upregulate antioxidant systems, including GPX4, SLC7A11, and NADPH-generating enzymes, thereby enhancing their capacity to neutralize lipid peroxidation [24]. In addition, dedifferentiation is associated with changes in membrane lipid composition, favoring MUFAs and reduced incorporation of PUFAs into phospholipids, which diminishes susceptibility to ferroptotic damage [85]. Importantly, dedifferentiation is not an all-or-none phenomenon but occurs along a spectrum, particularly during the evolution of radioiodine-refractory DTC. This continuum suggests that ferroptosis sensitivity may be dynamically regulated during disease progression, with intermediate states potentially representing windows of vulnerability to ferroptosis-inducing interventions.

### 4.3. Epigenetic and Post-Transcriptional Regulation of Ferroptosis Pathways

Beyond genetic alterations, epigenetic and post-transcriptional mechanisms play critical roles in shaping ferroptosis sensitivity in thyroid cancer. DNA methylation, histone modifications, and non-coding RNAs have been implicated in the regulation of key ferroptosis-related genes, including GPX4, SLC7A11, and ACSL4 [21,34,86]. MicroRNAs and long non-coding RNAs have emerged as modulators of ferroptosis-related pathways, influencing iron metabolism, lipid peroxidation, and antioxidant defense [87,88,89,90,91]. While many of these findings derive from in vitro or bioinformatic studies, they collectively suggest that ferroptosis regulation in DTC is embedded within broader epigenetic networks governing differentiation and stress responses [12,40,84,92]. Emerging evidence also implicates RNA modifications, such as *N*^6^-methyladenosine (m^6^A), in ferroptosis regulation across cancers, although data specific to DTC remain limited [93,94]. Given the growing recognition of epitranscriptomic regulation in thyroid tumor biology, this area represents an important frontier for future investigation.

### 4.4. Metabolic Rewiring and Ferroptosis Vulnerability

Metabolic reprogramming is a hallmark of thyroid cancer progression and a critical determinant of ferroptosis sensitivity. Alterations in mitochondrial metabolism, lipid synthesis, and redox cofactor availability collectively influence the balance between lipid peroxidation and antioxidant defense [27]. In DTC, shifts in glucose and lipid metabolism have been associated with disease aggressiveness and treatment resistance [95]. Increased flux through the pentose phosphate pathway and enhanced NADPH production support antioxidant defenses and ferroptosis resistance, while changes in fatty acid synthesis and desaturation modulate membrane susceptibility to lipid peroxidation [65]. Mitochondrial metabolism also contributes to ferroptosis regulation through pathways such as DHODH-mediated ubiquinol regeneration, which provides GPX4-independent protection against lipid peroxidation [50]. Although direct evidence in DTC is currently sparse, transcriptomic signatures suggest that mitochondrial ferroptosis defense pathways may be engaged in subsets of DTC, particularly those resistant to conventional therapies [34,96,97].

### 4.5. Clinical Implications and Conceptual Integration

Collectively, genetic drivers, epigenetic regulation, and metabolic rewiring converge to define ferroptosis sensitivity landscapes in DTC. Rather than acting as isolated determinants, these factors interact dynamically to shape tumor behavior under physiological and therapeutic stress. From a clinical perspective, this integrated view suggests that ferroptosis susceptibility may serve as a functional biomarker reflecting tumor differentiation status, oncogenic signaling, and metabolic state. Stratifying DTC patients based on ferroptosis-related features could inform therapeutic decision-making, particularly in the context of redifferentiation therapy, targeted kinase inhibition, and rational combination strategies aimed at overcoming resistance. In summary, ferroptosis in DTC is governed by a multilayered regulatory network encompassing genetic alterations, epigenetic modulation, and metabolic adaptation. Recognizing these determinants is essential for understanding intertumoral heterogeneity in ferroptosis sensitivity and for designing effective ferroptosis-based therapeutic approaches tailored to the biological context of DTC.

## 5. Lipid Metabolism and Membrane Remodeling in Differentiated Thyroid Cancer

Lipid metabolism has emerged as a central determinant of ferroptosis susceptibility, as the execution of ferroptotic cell death fundamentally depends on the peroxidation of PUFA-containing membrane phospholipids [7]. Cancer cells dynamically remodel lipid composition and membrane architecture to balance proliferative demands with protection against oxidative damage, thereby tuning their sensitivity to ferroptosis [98]. In DTC, lipid metabolic rewiring appears to be closely coupled to redox adaptation, differentiation status, and therapeutic pressure, positioning lipid metabolism as a critical axis of ferroptosis regulation.

### 5.1. PUFA Incorporation and Ferroptosis Execution

The susceptibility of cellular membranes to ferroptosis is largely dictated by the abundance and distribution of PUFA-containing phospholipids, which serve as substrates for lipid peroxidation [99]. ACSL4 plays a key role in esterifying PUFAs into phosphatidylethanolamines, thereby promoting ferroptotic vulnerability, whereas LPCAT3 facilitates their incorporation into membrane phospholipids [37]. Altered expression of ACSL4 and related lipid metabolic enzymes has been reported in both experimental and bioinformatic studies. Reduced ACSL4 expression and diminished PUFA incorporation have been associated with decreased lipid peroxidation and enhanced survival under oxidative stress, consistent with a ferroptosis-resistant phenotype [21,100]. These findings suggest that DTC cells may actively suppress PUFA-enriched membrane remodeling as a protective strategy, particularly during disease progression and under therapeutic pressure.

### 5.2. MUFA Synthesis and Protective Membrane Remodeling

In contrast to PUFAs, MUFAs are less susceptible to lipid peroxidation and can competitively displace PUFAs from membrane phospholipids, thereby conferring resistance to ferroptosis [39]. ACSL3-mediated MUFA incorporation has been identified as a key anti-ferroptotic mechanism across multiple cancer types. Emerging evidence indicates that thyroid cancer cells, especially those with more aggressive or dedifferentiated phenotypes, exhibit increased MUFA synthesis and incorporation into membrane lipids [101]. This shift in fatty acid composition reduces membrane peroxidizability and may represent an adaptive response to the intrinsically oxidative thyroid microenvironment. In DTC, gradual enrichment of MUFAs may accompany the transition toward radioiodine-refractory disease, linking lipid remodeling to loss of differentiation and therapeutic resistance [20,23].

### 5.3. Lipid Droplets as Buffers of Ferroptotic Stress

Lipid droplets (LDs) have traditionally been viewed as inert lipid storage organelles, but are now recognized as dynamic regulators of lipid homeostasis and redox balance [102]. By sequestering excess fatty acids and oxidizable lipids, LDs can limit substrate availability for lipid peroxidation and thereby attenuate ferroptosis [103]. Increased LD accumulation has been observed in more aggressive tumors and under metabolic stress conditions [101,104]. This phenomenon may serve as a buffering mechanism to protect membranes from ferroptotic damage, particularly in environments characterized by chronic oxidative stress. The mobilization of fatty acids between LDs and membranes thus represents a dynamic axis of ferroptosis regulation that may be exploited by DTC cells during progression and treatment adaptation [105].

### 5.4. Lipid Metabolic Reprogramming During Dedifferentiation and RAIR-DTC

Dedifferentiation in thyroid cancer is accompanied by profound metabolic changes, including shifts in lipid synthesis, desaturation, and storage [106]. As tumors lose thyroid-specific functions and iodine avidity, they increasingly rely on alternative metabolic pathways to sustain survival under stress [95]. Lipid metabolic rewiring is a prominent feature of this transition. Several studies have demonstrated that dedifferentiated thyroid cancer cells display reduced lipid peroxidation capacity and enhanced antioxidant buffering, consistent with ferroptosis resistance [19,107]. These changes may reflect selective pressure to minimize lipid-derived oxidative damage in the context of chronic redox stress and therapeutic exposure [108]. Importantly, the gradual nature of dedifferentiation suggests that lipid remodeling is an evolving process, potentially creating transient states of vulnerability that could be targeted therapeutically [109].

### 5.5. Crosstalk Between Lipid Metabolism and Redox Signaling

Lipid metabolism in DTC does not operate in isolation but is tightly integrated with redox signaling pathways, including Nrf2-mediated antioxidant responses [57,58]. Nrf2 activation promotes not only antioxidant gene expression but also lipid metabolic programs that favor ferroptosis resistance, such as MUFA synthesis and LD formation [52]. Nrf2-driven lipid remodeling may be particularly advantageous, given the organ’s intrinsic oxidative environment [73]. This coordinated regulation enables cancer cells to fine-tune membrane composition and redox balance, thereby maintaining viability under both physiological and therapeutic oxidative stress [110]. Disrupting this crosstalk between lipid metabolism and redox signaling could therefore sensitize DTC cells to ferroptosis-inducing strategies [56,57]. These observations highlight that lipid metabolism is not an isolated determinant of ferroptosis but operates in concert with redox signaling and antioxidant systems. In DTC, Nrf2-driven antioxidant programs, GPX4 activity, and lipid remodeling collectively establish a coordinated defense against lipid peroxidation, reinforcing ferroptosis resistance in a context-dependent manner.

### 5.6. Therapeutic Implications of Lipid Remodeling in DTC

The central role of lipid metabolism in ferroptosis regulation has significant translational implications for DTC. Targeting enzymes involved in PUFA incorporation, MUFA synthesis, or LD dynamics could reprogram membrane susceptibility to lipid peroxidation and restore ferroptosis sensitivity [24]. Moreover, lipid metabolic states may serve as biomarkers for stratifying patients likely to benefit from ferroptosis-based interventions. Importantly, therapeutic strategies aimed at redifferentiation or targeted kinase inhibition may inadvertently alter lipid metabolism, thereby reshaping ferroptosis vulnerability [15]. Understanding these interactions will be essential for designing rational combination therapies that exploit metabolic liabilities without exacerbating resistance.

### 5.7. Conceptual Integration

In summary, lipid metabolism and membrane remodeling constitute a central axis governing ferroptosis sensitivity in DTC. Through coordinated regulation of fatty acid composition, lipid storage, and membrane architecture, DTC cells dynamically modulate their susceptibility to lipid peroxidation and ferroptotic death. In summary, lipid metabolism and membrane remodeling constitute a central axis of ferroptosis regulation in DTC, linking redox adaptation with differentiation status and therapeutic pressure.

## 6. Tumor Microenvironment and Immune Interactions: Double-Edged Roles of Ferroptosis in Differentiated Thyroid Cancer

The tumor microenvironment (TME) is increasingly recognized as a critical determinant of tumor progression, therapeutic response, and resistance in DTC [111]. Beyond cancer cell–intrinsic signaling, interactions between tumor cells, immune infiltrates, stromal components, and metabolic factors shape disease behavior. Ferroptosis, through its unique biochemical features and immunomodulatory consequences, has emerged as a key interface between cancer cell death and the tumor microenvironment [112]. Unlike apoptosis, ferroptosis is characterized by extensive lipid peroxidation and release of oxidized lipid species, which can profoundly influence surrounding immune and stromal cells [113]. In the context of DTC, where immune checkpoint inhibitors (ICIs) efficacy remains limited and immune landscapes are heterogeneous, understanding how ferroptosis shapes the TME is of particular relevance [114]. However, it should be noted that much of the current understanding of ferroptosis–immune interactions is derived from non-thyroid cancer models. Direct mechanistic evidence in DTC remains limited. Therefore, several proposed interactions within the thyroid tumor microenvironment should be considered hypothesis-generating rather than definitively established.

### 6.1. Ferroptotic Signaling and Immunogenic Consequences

Ferroptotic cell death can generate a complex array of damage-associated molecular patterns (DAMPs), lipid peroxidation products, and oxidized phospholipids that act as immunomodulatory signals [115]. These signals may promote immune cell recruitment and activation under certain conditions, while fostering immunosuppression or tolerance in others, underscoring the dual nature of ferroptosis within the TME [8]. In thyroid cancer models, ferroptosis-related gene expression patterns have been associated with differential immune infiltration, including variations in macrophage polarization and T-cell abundance [84,86,88,116]. These observations suggest that ferroptosis is not merely a terminal event but an active participant in shaping immune contexture.

### 6.2. Macrophages and Ferroptosis in DTC

Tumor-associated macrophages (TAMs) represent a dominant immune population in the thyroid cancer microenvironment and play pivotal roles in tumor growth, angiogenesis, and immune regulation [117]. Ferroptosis has been shown to influence macrophage behavior through the release of oxidized lipids and iron-containing molecules, which can modulate macrophage polarization and function [118]. In DTC, bioinformatic studies have linked ferroptosis-related signatures to macrophage-enriched immune landscapes, often associated with poorer prognosis [11,86]. Ferroptotic stress may promote an immunosuppressive, pro-tumorigenic macrophage phenotype under chronic conditions, thereby facilitating tumor progression rather than immune-mediated clearance [119,120]. This phenomenon may be particularly relevant in RAIR-DTC, where prolonged oxidative stress and therapy-induced damage create a permissive environment for macrophage-driven immune suppression [117]. However, most of these observations are based on correlative or transcriptomic analyses rather than direct functional studies in DTC. Thus, the causal relationship between ferroptosis and macrophage polarization remains to be established.

### 6.3. T Cells, Immune Evasion, and Ferroptosis Resistance

T-cell infiltration and activation are key determinants of immunotherapy response; however, DTC generally exhibit modest T-cell infiltration and limited responsiveness to immune checkpoint blockade [121]. Recent studies suggest that ferroptosis-related metabolic and redox adaptations may contribute to immune evasion in DTC. Enhanced antioxidant capacity and altered lipid metabolism in ferroptosis-resistant tumor cells can reduce immunogenic lipid peroxidation products and dampen immune activation [122,123]. Moreover, iron and lipid metabolites released during ferroptotic stress may impair T-cell function or survival, further reinforcing immune escape mechanisms [124,125]. These interactions highlight a potential feedback loop in which ferroptosis resistance and immune evasion are mutually reinforcing in DTC. Nevertheless, direct evidence linking ferroptosis-induced metabolic changes to T-cell dysfunction in DTC is currently lacking, and these interactions should be interpreted with caution.

### 6.4. Stromal and Vascular Components of the TME

Beyond immune cells, stromal fibroblasts and endothelial cells contribute to the ferroptosis landscape within the TME. Cancer-associated fibroblasts (CAFs) can modulate redox balance and lipid availability through metabolic crosstalk, potentially influencing ferroptosis susceptibility in neighboring tumor cells [126]. Similarly, vascular alterations and hypoxic gradients affect iron metabolism, lipid oxidation, and antioxidant capacity, indirectly shaping ferroptotic vulnerability [127]. Although direct evidence in DTC remains limited, transcriptomic analyses suggest that ferroptosis-related gene expression correlates with stromal activation and angiogenic signatures in thyroid cancer cohorts [34,116]. These findings suggest a broader role for the TME in regulating ferroptosis beyond cancer cell–intrinsic mechanisms.

### 6.5. Ferroptosis, Chronic Inflammation, and Therapy Resistance

Chronic inflammation is a hallmark of the thyroid tumor microenvironment and has been implicated in tumor progression and resistance to therapy [128,129]. Ferroptosis may contribute to this inflammatory milieu by releasing oxidized lipids and iron, which can perpetuate inflammatory signaling and oxidative stress [130]. In DTC, prolonged exposure to sublethal oxidative stress—whether driven by intrinsic redox biology or therapeutic interventions—may favor the emergence of ferroptosis-resistant clones while sustaining a pro-tumorigenic inflammatory environment [14,24]. This scenario aligns with observations that ferroptosis-related gene signatures are associated with adverse clinical outcomes and immune suppression in DTC cohorts [11,12,92].

### 6.6. Implications for Immunotherapy and Combination Strategies

The complex interplay between ferroptosis and the TME has important implications for therapeutic development. While inducing ferroptosis may enhance tumor cell killing, unrestrained ferroptotic signaling could also promote immunosuppressive or inflammatory conditions that undermine long-term disease control [131]. Thus, ferroptosis-based therapies must be carefully calibrated within the immune context of DTC. Rational combination strategies that integrate ferroptosis modulation with immunotherapy, targeted therapy, or redifferentiation approaches may offer a means to harness the beneficial aspects of ferroptosis while mitigating its potential adverse effects on the TME. Identifying biomarkers that reflect both ferroptosis susceptibility and immune landscape will be essential for patient stratification and treatment optimization.

### 6.7. Conceptual Synthesis

In summary, ferroptosis occupies a multifaceted role within the DTC microenvironment, acting as both a tumor-suppressive and tumor-promoting force depending on context. Through its impact on immune cells, stromal components, and inflammatory signaling, ferroptosis extends beyond cancer cell death to shape the broader ecosystem in which thyroid tumors evolve. Recognizing this duality is critical for interpreting experimental findings and for designing ferroptosis-targeted interventions that achieve durable therapeutic benefit in DTC. This microenvironmental perspective sets the stage for the subsequent section, which will explore translational strategies for targeting ferroptosis in differentiated and radioiodine-refractory thyroid cancer.

## 7. Translational Implications: Targeting Ferroptosis in Differentiated and Radioiodine-Refractory Thyroid Cancer

**Figure 3:**
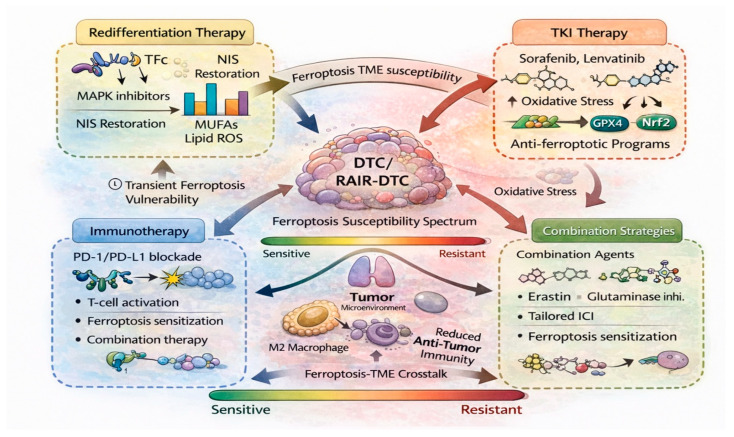
**Translational integration of ferroptosis targeting in differentiated and radioiodine-refractory thyroid cancer.** This schematic illustrates a translational framework for incorporating ferroptosis modulation into current therapeutic strategies for DTC and radioactive iodine-refractory DTC (RAIR-DTC). At the center, thyroid cancer cells are positioned along a spectrum of ferroptosis susceptibility that evolves with differentiation status and therapeutic pressure. Redifferentiation therapies targeting oncogenic MAPK signaling may transiently restore iodine handling and reshape redox and lipid metabolic states, creating windows of increased ferroptosis vulnerability. Targeted kinase inhibitors, such as sorafenib and lenvatinib, induce oxidative stress but frequently trigger adaptive anti-ferroptotic programs mediated by GPX4, Nrf2, and lipid remodeling. Ferroptosis also interacts bidirectionally with the tumor microenvironment, influencing macrophage polarization and immune activity, which may limit the efficacy of immune checkpoint inhibition in unselected DTC. Combination strategies integrating ferroptosis sensitization with redifferentiation therapy, targeted agents, or immunotherapy are proposed to overcome resistance and enhance therapeutic responses in advanced and radioiodine-refractory thyroid cancer. Abbreviations: DTC, differentiated thyroid cancer; GPX4, glutathione peroxidase 4; ICI, immune checkpoint inhibitor; MAPK, mitogen-activated protein kinase; MUFAs, monounsaturated fatty acids; NIS, sodium/iodide symporter; Nrf2, nuclear-factor-erythroid-2-related factor 2; PD-1, programmed cell death protein 1; PD-L1, programmed death-ligand 1; RAIR-DTC, radioiodine-refractory differentiated thyroid cancer; TKI, tyrosine kinase inhibitor; TME, tumor microenvironment.

Despite generally favorable outcomes, a subset of patients with DTC develop progressive, metastatic, or radioiodine-refractory disease that remains challenging to treat [23]. In this setting, ferroptosis has emerged not only as a mechanistic vulnerability but also as a promising conceptual framework, supported primarily by preclinical evidence (Figure 3). Translating ferroptosis biology into clinical benefit requires careful consideration of disease stage, differentiation status, and existing treatment paradigms (Table 2).

### 7.1. Ferroptosis and Redifferentiation Therapy in RAIR-DTC

Loss of radioiodine avidity represents a defining feature of RAIR-DTC and reflects profound dedifferentiation driven primarily by aberrant MAPK signaling [1,23]. Redifferentiation strategies using MAPK pathway inhibitors have demonstrated partial restoration of NIS expression and radioiodine uptake in selected patients, yet clinical responses remain variable and often transient [81,132]. From a ferroptosis perspective, MAPK inhibition may exert dual effects. On one hand, suppression of oncogenic MAPK signaling can attenuate antioxidant and metabolic defense programs associated with ferroptosis resistance, potentially increasing susceptibility to lipid-peroxidation-driven cell death [82]. On the other hand, restoration of thyroid-specific functions may reintroduce physiological redox processes that reshape ferroptosis sensitivity in complex ways [24]. These opposing effects underscore the need to view ferroptosis and redifferentiation as interconnected, rather than independent, therapeutic axes [17]. Preclinical studies suggest that combining redifferentiation therapy with ferroptosis-sensitizing approaches could enhance therapeutic efficacy by exploiting transient vulnerabilities during metabolic and redox reprogramming [20,21]. Supporting this concept, recent preclinical studies have demonstrated that combined targeting of BRAF^V600E^ signaling and ferroptosis pathways results in synergistic antitumor effects through enhanced oxidative stress and iron-dependent lipid peroxidation. Such strategies may be particularly relevant in RAIR-DTC, where conventional cytotoxic approaches have limited effectiveness.

### 7.2. Targeted Kinase Inhibitors and Ferroptosis Modulation

Multikinase inhibitors such as sorafenib and lenvatinib remain standard systemic therapies for progressive RAIR-DTC, yet their clinical benefit is often limited by toxicity and acquired resistance [133,134]. Notably, several targeted agents used in thyroid cancer have been shown to intersect with ferroptosis-related pathways, suggesting that ferroptosis may contribute to their antitumor activity. Sorafenib, in particular, has been reported to induce ferroptosis through inhibition of system Xc^−^ and depletion of intracellular glutathione in various cancer models [135]. In thyroid cancer cells, sorafenib-induced oxidative stress and lipid peroxidation have been linked to growth suppression, although adaptive antioxidant responses frequently limit durable efficacy [136]. Lenvatinib has similarly been associated with redox perturbations and metabolic stress, raising the possibility that ferroptosis contributes to its cytostatic effects [137]. Resistance to targeted therapy in DTC is increasingly associated with reinforcement of ferroptosis defense mechanisms, including upregulation of GPX4, SLC7A11, and lipid remodeling pathways [44,71]. These observations suggest that ferroptosis resistance may represent a convergent escape mechanism following prolonged kinase inhibition, highlighting the rationale for combination strategies that disrupt anti-ferroptotic adaptations.

### 7.3. Ferroptosis-Based Combination Strategies

Given the multifactorial regulation of ferroptosis in DTC, monotherapy approaches targeting single ferroptosis regulators are unlikely to achieve sustained clinical benefit. Instead, rational combination strategies tailored to disease context may offer potential translational value, although these strategies remain to be validated in clinical settings. Potential combination paradigms include ferroptosis sensitization alongside targeted kinase inhibitors to prevent or delay resistance, integration with redifferentiation therapy to exploit transient metabolic vulnerabilities, and modulation of ferroptosis in conjunction with radiotherapy or immunotherapy [18,41,138,139]. Importantly, such strategies must account for the thyroid-specific redox environment and the risk of excessive oxidative damage to surrounding normal tissues. Nanoparticle-based delivery systems and tumor-targeted approaches have been proposed as means to enhance ferroptosis induction while limiting systemic toxicity [140]. Emerging experimental data suggest that co-targeting ferroptosis pathways alongside kinase inhibition or redifferentiation therapy may enhance treatment response and delay resistance, although these findings remain to be validated in clinical settings. Although clinical data in thyroid cancer are currently lacking, these platforms may facilitate selective modulation of ferroptosis pathways in RAIR-DTC.

### 7.4. Ferroptosis and Immunotherapy Sensitization

ICIs have shown limited activity in unselected DTC populations, reflecting low tumor mutational burden and immunologically “cold” tumor microenvironments [141]. Ferroptosis has been proposed as a potential means to enhance tumor immunogenicity through the release of oxidized lipid signals and modulation of immune cell recruitment [16]. However, the immunological consequences of ferroptosis are context-dependent and may promote either immune activation or suppression [142]. In DTC, where chronic oxidative stress and macrophage-dominated immune landscapes are common, indiscriminate induction of ferroptosis could exacerbate immunosuppressive signaling [111,117]. Thus, careful integration of ferroptosis modulation with immunotherapy will require biomarker-guided approaches and a nuanced understanding of TME dynamics [143,144].

### 7.5. Biomarkers and Patient Stratification for Ferroptosis-Based Therapy

A major barrier to clinical translation of ferroptosis-targeted strategies is the lack of validated biomarkers to identify patients most likely to benefit. Multiple studies have proposed ferroptosis-related gene signatures associated with prognosis, immune infiltration, and therapy response in thyroid cancer cohorts [12,34,86,92]. While promising, these signatures require prospective validation and functional correlation. Potential biomarker candidates include expression levels of GPX4, SLC7A11, ACSL4, and Nrf2 target genes, as well as lipid metabolic profiles reflecting membrane susceptibility to peroxidation [47,56,58,100,145].

Integration of ferroptosis biomarkers with established clinical parameters, such as differentiation status and RAI avidity, may enable more precise patient stratification and therapeutic decision-making. From a clinical standpoint, most currently proposed ferroptosis-related biomarkers in DTC are derived from tumor tissue analyses, including immunohistochemistry or transcriptomic profiling of surgical specimens. While these approaches provide mechanistic insight, their applicability in routine clinical decision-making is limited, particularly in advanced or inoperable cases. In contrast, liquid-biopsy-based approaches, such as circulating tumor DNA (ctDNA), circulating RNA, or lipidomic profiling, may offer a minimally invasive means to assess ferroptosis-related states. However, such strategies remain largely unexplored in DTC and require further validation. Importantly, the sensitivity and specificity of currently proposed ferroptosis-related biomarkers remain unclear. Most reported signatures are derived from retrospective datasets and have not been validated across independent cohorts or prospective clinical settings. Furthermore, overlap with general oxidative stress or metabolic markers may limit their specificity for ferroptosis per se.

From a translational perspective, clinically actionable ferroptosis biomarkers in DTC will likely require integration of multiple parameters rather than reliance on single markers. Composite approaches combining gene expression (e.g., GPX4/SLC7A11), lipid metabolic profiles, and redox-related signatures may better capture ferroptosis susceptibility. In addition, aligning biomarker development with clinically relevant contexts—such as predicting response to redifferentiation therapy, kinase inhibitors, or combination strategies—will be critical for their practical implementation. At present, the lack of standardized assays, limited prospective validation, and uncertainty regarding optimal sampling methods represent major barriers to clinical implementation of ferroptosis-related biomarkers in DTC.

### 7.6. Clinical Perspective and Future Directions

From a translational standpoint, ferroptosis should be viewed not as a stand-alone therapeutic target but as a modulatory axis that intersects with existing treatment strategies in differentiated and RAIR-DTC. The greatest clinical value of ferroptosis modulation may lie in its ability to sensitize tumors, pending further clinical validation to established therapies, overcome resistance, and reshape tumor–immune interactions [22]. Future clinical studies will need to address key questions, including optimal timing of ferroptosis-targeted interventions, identification of predictive biomarkers, and management of potential toxicities related to redox imbalance [138,146]. Advances in patient-derived models, molecular imaging, and lipidomics are likely to accelerate progress in this area. In particular, prospective studies integrating ferroptosis biomarkers with therapeutic outcomes will be critical to bridge the gap between experimental findings and clinical application.

### 7.7. Conceptual Synthesis

In summary, ferroptosis represents a promising but still largely preclinical therapeutic opportunity in DTC. By integrating ferroptosis biology with redifferentiation strategies, targeted therapy, and immune modulation, it may be possible to develop more effective and durable treatment paradigms for RAIR-DTC. Realizing this potential will require careful alignment of mechanistic insights with clinical realities, emphasizing context-dependent application rather than universal induction of ferroptotic cell death.

## 8. Conclusions and Future Perspectives

Ferroptosis has emerged as a critical regulatory node at the intersection of redox biology, lipid metabolism, and cell fate determination in cancer, and DTC represents a particularly compelling context in which to interrogate this pathway. Unlike many malignancies in which oxidative stress is primarily a consequence of oncogenic metabolism, thyroid cancer develops within an organ intrinsically shaped by iodine-dependent redox reactions, conferring unique constraints and opportunities for ferroptosis regulation [61]. Mechanistically, ferroptosis in DTC is best understood as the product of an integrated regulatory network involving redox signaling, antioxidant capacity, and lipid metabolic remodeling, rather than as a pathway governed by isolated molecular components. This organ-specific biology fundamentally influences how ferroptosis is engaged, resisted, or exploited during thyroid tumor progression.

In this review, we have highlighted that ferroptosis in DTC should not be conceptualized as a uniform or binary cell death mechanism. Instead, ferroptosis susceptibility is dynamically regulated by differentiation status, oncogenic signaling, lipid metabolic remodeling, and adaptive antioxidant programs. As tumors transition from iodine-avid differentiated states to radioiodine-refractory disease, progressive reinforcement of ferroptosis resistance emerges as a hallmark of therapeutic adaptation, closely intertwined with dedifferentiation and redox reprogramming. This perspective reframes ferroptosis not merely as a cytotoxic endpoint, but as a functional readout of tumor state and evolutionary trajectory. Despite recent advances, several critical questions remain unresolved. It is still unclear how ferroptosis susceptibility is dynamically regulated during the transition from differentiated to radioiodine-refractory states, and whether this process creates predictable therapeutic windows. In addition, the precise role of thyroid-specific iodine metabolism in modulating ferroptosis sensitivity has not been fully elucidated. Furthermore, the causal relationships between ferroptosis and immune modulation within the DTC tumor microenvironment remain incompletely defined.

Importantly, ferroptosis also extends beyond cancer cell–intrinsic processes to shape the tumor microenvironment and immune landscape. Ferroptotic signaling influences macrophage behavior, inflammatory tone, and immune exclusion, underscoring its dual capacity to suppress or support tumor progression depending on context. In DTC, where immune checkpoint blockade has shown limited efficacy, understanding how ferroptosis intersects with immune regulation may be essential for developing effective combination strategies rather than indiscriminate ferroptosis induction. From a translational standpoint, the greatest promise of ferroptosis in DTC lies in its potential role as a therapeutic sensitizer rather than a stand-alone target. Integration of ferroptosis modulation with redifferentiation therapy, targeted kinase inhibition, radiotherapy, or immunotherapy offers a rational framework for overcoming resistance in radioiodine-refractory disease. However, such approaches must be carefully tailored to disease stage, differentiation status, and metabolic context to avoid unintended promotion of immune suppression or tissue toxicity. A major challenge for the field remains the identification of robust biomarkers that capture ferroptosis susceptibility in clinically meaningful ways. While ferroptosis-related gene signatures and lipid metabolic profiles have shown promise in retrospective analyses, prospective validation and functional correlation are urgently needed before clinical implementation. Incorporating ferroptosis biomarkers into existing risk stratification frameworks may ultimately enable more precise patient selection and personalized therapeutic strategies.

Looking forward, several priorities emerge for future research. First, studies should focus on ferroptosis regulation in clinically relevant models of differentiated and radioiodine-refractory thyroid cancer, including patient-derived organoids and in vivo systems that recapitulate thyroid-specific redox environments. Second, mechanistic dissection of how redifferentiation therapies reshape ferroptosis susceptibility could reveal transient vulnerabilities exploitable by combination treatment. Third, careful evaluation of ferroptosis–immune interactions will be essential for safely integrating ferroptosis modulation with immunotherapeutic approaches. Addressing these unresolved questions will be essential for translating ferroptosis biology into clinically meaningful strategies in DTC.

In conclusion, ferroptosis represents a context-dependent vulnerability shaped by thyroid-specific redox biology and tumor evolution. By reframing ferroptosis as a modulatory axis linked to dedifferentiation and treatment resistance, rather than a universal death pathway, this review provides a conceptual roadmap for future mechanistic studies and translational efforts aimed at improving outcomes for patients with advanced and radioiodine-refractory thyroid cancer.

## Figures and Tables

**Table 1:** Comparison of core ferroptosis features and thyroid-specific adaptations across differentiation states.

Biological Context	Key Thyroid-Specific Features	Ferroptosis-Related Alterations	Representative Regulators/Pathways	Biological Implication
Normal thyroid follicular cells	Physiological iodine oxidation, chronic H_2_O_2_ production	Balanced lipid peroxidation and antioxidant buffering	DUOX, GPX4, GSH system	Physiological redox tolerance
Differentiated thyroid cancer (PTC/FTC)	Retained thyroid-specific gene expression	Context-dependent ferroptosis susceptibility	GPX4, SLC7A11, ACSL4	Conditional vulnerability
Dedifferentiating DTC	Loss of NIS, metabolic rewiring	Reinforcement of anti-ferroptotic defenses	Nrf2 activation, MUFA synthesis	Emerging resistance
Radioiodine-refractory DTC	Absent iodine uptake, therapy-induced stress	Marked ferroptosis resistance	GPX4↑, lipid droplet accumulation	Therapy resistance and survival

**Table 2:** Translational implications of ferroptosis targeting in differentiated and RAIR thyroid cancer.

Clinical Context	Therapeutic Setting	Ferroptosis-Related Mechanism	Evidence Level	Translational Implication	Clinical Context
RAIR-DTC	MAPK-inhibition-based redifferentiation	Redox and lipid remodeling alters ferroptosis sensitivity	Preclinical, translational	Combination strategy opportunity	RAIR-DTC
Progressive DTC	TKI therapy (sorafenib, lenvatinib)	Therapy-induced oxidative stress intersects with ferroptosis	Experimental	Sensitization to delay resistance	Progressive DTC
Therapy-resistant DTC	Acquired resistance	Reinforcement of anti-ferroptotic programs	Experimental, bioinformatic	Targeting resistance nodes	Therapy-resistant DTC
Immune-cold DTC	Immunotherapy	Ferroptosis–TME crosstalk	Mechanistic	Immune modulation	Immune-cold DTC

## Data Availability

No new data were created or analyzed in this study.

## References

[1] Ringel M.D., Sosa J.A., Baloch Z., Bischoff L., Bloom G., Brent G.A., Brock P.L., Chou R., Flavell R.R., Goldner W. (2025). 2025 American Thyroid Association Management Guidelines for Adult Patients with Differentiated Thyroid Cancer. Thyroid.

[2] Gianoukakis A.G., Choe J.H., Bowles D.W., Brose M.S., Wirth L.J., Owonikoko T., Babajanyan S., Worden F.P. (2024). Real-world practice patterns and outcomes for RAI-refractory differentiated thyroid cancer. Eur. Thyroid J..

[3] Chen P., Yao Y., Tan H., Li J. (2024). Systemic treatments for radioiodine-refractory thyroid cancers. Front. Endocrinol..

[4] Bulotta S., Celano M., Costante G., Russo D. (2020). Novel therapeutic options for radioiodine-refractory thyroid cancer: Redifferentiation and beyond. Curr. Opin. Oncol..

[5] Dixon S.J., Lemberg K.M., Lamprecht M.R., Skouta R., Zaitsev E.M., Gleason C.E., Patel D.N., Bauer A.J., Cantley A.M., Yang W.S. (2012). Ferroptosis: An iron-dependent form of nonapoptotic cell death. Cell.

[6] Stockwell B.R., Friedmann Angeli J.P., Bayir H., Bush A.I., Conrad M., Dixon S.J., Fulda S., Gascón S., Hatzios S.K., Kagan V.E. (2017). Ferroptosis: A Regulated Cell Death Nexus Linking Metabolism, Redox Biology, and Disease. Cell.

[7] Liang D., Minikes A.M., Jiang X. (2022). Ferroptosis at the intersection of lipid metabolism and cellular signaling. Mol. Cell.

[8] Lei G., Zhuang L., Gan B. (2024). The roles of ferroptosis in cancer: Tumor suppression, tumor microenvironment, and therapeutic interventions. Cancer Cell.

[9] Kochman J., Jakubczyk K., Bargiel P., Janda-Milczarek K. (2021). The Influence of Oxidative Stress on Thyroid Diseases. Antioxidants.

[10] Ameziane El Hassani R., Buffet C., Leboulleux S., Dupuy C. (2019). Oxidative stress in thyroid carcinomas: Biological and clinical significance. Endocr. Relat. Cancer.

[11] Ren X., Du H., Cheng W., Wang Y., Xu Y., Yan S., Gao Y. (2022). Construction of a ferroptosis-related eight gene signature for predicting the prognosis and immune infiltration of thyroid cancer. Front. Endocrinol..

[12] Wang Y., Yang J., Chen S., Wang W., Teng L. (2022). Identification and Validation of a Prognostic Signature for Thyroid Cancer Based on Ferroptosis-Related Genes. Genes.

[13] Ma Y.Y., Zhou W.Y., Qian Y., Mu Y.Y., Zhang W. (2024). SOX13 as a potential prognostic biomarker linked to immune infiltration and ferroptosis inhibits the proliferation, migration, and metastasis of thyroid cancer cells. Front. Immunol..

[14] Sekhar K.R., Cyr S., Baregamian N. (2023). Ferroptosis Inducers in Thyroid Cancer. World J. Surg..

[15] Liu X., Wang L., Xi X., Zhou T., Sun Z., Zhang B. (2025). Targeting ferroptosis: A novel insight into thyroid cancer therapy. Front. Endocrinol..

[16] Yin L., Luo X., Zhang X., Cheng B. (2024). The evolving process of ferroptosis in thyroid cancer: Novel mechanisms and opportunities. J. Cell Mol. Med..

[17] Lee J., Roh J.L. (2025). Ferroptosis in Anaplastic Thyroid Cancer: Molecular Mechanisms, Preclinical Evidence, and Therapeutic Prospects. Cells.

[18] Hu J., Ghosh C., Khaket T.P., Yang Z., Tabdili Y., Alamaw E.D., Boufraqech M., Dixon S.J., Kebebew E. (2026). Dual targeting of BRAF(V600E) and ferroptosis results in synergistic anticancer activity via iron overload and enhanced oxidative stress. J. Exp. Clin. Cancer Res..

[19] Dai B., Li J., Xu L., Chen W., Chen J., Song M., Liu Y., Wang L., Zhang L., Chen J. (2026). YTHDF2-mediated stabilization of SREBF1 promotes lipid metabolic reprogramming and ferroptosis-associated radioresistance in anaplastic thyroid carcinoma. Cancer Lett..

[20] He Y., Tang Z., Xu M., Huang T. (2025). Dedifferentiation and Redifferentiation of Follicular-Cell-Derived Thyroid Carcinoma: Mechanisms and Therapeutic Implications. Biomedicines.

[21] Chen Y., Pan G., Wu F., Zhang Y., Li Y., Luo D. (2024). Ferroptosis in thyroid cancer: Potential mechanisms, effective therapeutic targets and predictive biomarker. Biomed. Pharmacother..

[22] Zhou Q., Meng Y., Li D., Yao L., Le J., Liu Y., Sun Y., Zeng F., Chen X., Deng G. (2024). Ferroptosis in cancer: From molecular mechanisms to therapeutic strategies. Signal Transduct. Target. Ther..

[23] Shen H., Zhu R., Liu Y., Hong Y., Ge J., Xuan J., Niu W., Yu X., Qin J.J., Li Q. (2024). Radioiodine-refractory differentiated thyroid cancer: Molecular mechanisms and therapeutic strategies for radioiodine resistance. Drug Resist. Updates.

[24] Tian W., Su X., Hu C., Chen D., Li P. (2025). Ferroptosis in thyroid cancer: Mechanisms, current status, and treatment. Front. Oncol..

[25] Lei G., Zhuang L., Gan B. (2022). Targeting ferroptosis as a vulnerability in cancer. Nat. Rev. Cancer.

[26] Guo F., Zong S., Zhang X., Ren Z., Shao H., Li J., Wang X., Li Y., Wang X., Chen K. (2026). Ferroptosis and metastasis: Molecular checkpoints, microenvironmental dynamics, and therapeutic opportunities. Mol. Cancer.

[27] Alves F., Lane D., Nguyen T.P.M., Bush A.I., Ayton S. (2025). In defence of ferroptosis. Signal Transduct. Target. Ther..

[28] Tang D., Kang R. (2024). NFE2L2 and ferroptosis resistance in cancer therapy. Cancer Drug Resist..

[29] Shi Z.D., Pang K., Wu Z.X., Dong Y., Hao L., Qin J.X., Wang W., Chen Z.S., Han C.H. (2023). Tumor cell plasticity in targeted therapy-induced resistance: Mechanisms and new strategies. Signal Transduct. Target. Ther..

[30] Liang Y., Chen W.M., Zhang Y., Li L. (2026). Remodeling the tumor dormancy ecosystem to prevent recurrence and metastasis. Signal Transduct. Target. Ther..

[31] Stoyanovsky D.A., Tyurina Y.Y., Shrivastava I., Bahar I., Tyurin V.A., Protchenko O., Jadhav S., Bolevich S.B., Kozlov A.V., Vladimirov Y.A. (2019). Iron catalysis of lipid peroxidation in ferroptosis: Regulated enzymatic or random free radical reaction?. Free Radic. Biol. Med..

[32] Ru Q., Li Y., Chen L., Wu Y., Min J., Wang F. (2024). Iron homeostasis and ferroptosis in human diseases: Mechanisms and therapeutic prospects. Signal Transduct. Target. Ther..

[33] Jin T., Ge L., Chen J., Wang W., Zhang L., Ge M. (2023). Identification of iron metabolism-related genes as prognostic indicators for papillary thyroid carcinoma: A retrospective study. PeerJ.

[34] Qian X., Tang J., Li L., Chen Z., Chen L., Chu Y. (2021). A new ferroptosis-related gene model for prognostic prediction of papillary thyroid carcinoma. Bioengineered.

[35] Sun K., Li C., Liao S., Yao X., Ouyang Y., Liu Y., Wang Z., Li Z., Yao F. (2022). Ferritinophagy, a form of autophagic ferroptosis: New insights into cancer treatment. Front. Pharmacol..

[36] Mortensen M.S., Ruiz J., Watts J.L. (2023). Polyunsaturated Fatty Acids Drive Lipid Peroxidation during Ferroptosis. Cells.

[37] Doll S., Proneth B., Tyurina Y.Y., Panzilius E., Kobayashi S., Ingold I., Irmler M., Beckers J., Aichler M., Walch A. (2017). ACSL4 dictates ferroptosis sensitivity by shaping cellular lipid composition. Nat. Chem. Biol..

[38] Lagrost L., Masson D. (2022). The expanding role of lyso-phosphatidylcholine acyltransferase-3 (LPCAT3), a phospholipid remodeling enzyme, in health and disease. Curr. Opin. Lipidol..

[39] Magtanong L., Ko P.J., To M., Cao J.Y., Forcina G.C., Tarangelo A., Ward C.C., Cho K., Patti G.J., Nomura D.K. (2019). Exogenous Monounsaturated Fatty Acids Promote a Ferroptosis-Resistant Cell State. Cell Chem. Biol..

[40] Ru Z., Li S., Wang M., Ni Y., Qiao H. (2025). Exploring Immune-Related Ferroptosis Genes in Thyroid Cancer: A Comprehensive Analysis. Biomedicines.

[41] Hu C., Tian W., Tang Y., Wang P., Kuan T., Wei W., Chen D., Li P., Su X. (2026). USP7 inhibitor P5091 enhances the antitumor efficacy of vemurafenib in BRAF(V600E)-mutant thyroid cancer via ferroptosis. Biochem. Pharmacol..

[42] Lee J., Roh J.L. (2023). Targeting GPX4 in human cancer: Implications of ferroptosis induction for tackling cancer resilience. Cancer Lett..

[43] Sekhar K.R., Hanna D.N., Cyr S., Baechle J.J., Kuravi S., Balusu R., Rathmell K., Baregamian N. (2022). Glutathione peroxidase 4 inhibition induces ferroptosis and mTOR pathway suppression in thyroid cancer. Sci. Rep..

[44] Lian F., Dong D., Pu J., Yang G., Yang J., Yang S., Wang Y., Zhao B., Lu M. (2024). Ubiquitin-specific peptidase 10 attenuates the ferroptosis to promote thyroid cancer malignancy by facilitating GPX4 via elevating SIRT6. Environ. Toxicol..

[45] Ji F.H., Fu X.H., Li G.Q., He Q., Qiu X.G. (2022). FTO Prevents Thyroid Cancer Progression by SLC7A11 m6A Methylation in a Ferroptosis-Dependent Manner. Front. Endocrinol..

[46] Wang L., Zhang Y., Yang J., Liu L., Yao B., Tian Z., He J. (2021). The Knockdown of ETV4 Inhibits the Papillary Thyroid Cancer Development by Promoting Ferroptosis Upon SLC7A11 Downregulation. DNA Cell Biol..

[47] Chen H., Peng F., Xu J., Wang G., Zhao Y. (2023). Increased expression of GPX4 promotes the tumorigenesis of thyroid cancer by inhibiting ferroptosis and predicts poor clinical outcomes. Aging.

[48] Bersuker K., Hendricks J.M., Li Z., Magtanong L., Ford B., Tang P.H., Roberts M.A., Tong B., Maimone T.J., Zoncu R. (2019). The CoQ oxidoreductase FSP1 acts parallel to GPX4 to inhibit ferroptosis. Nature.

[49] Kraft V.A.N., Bezjian C.T., Pfeiffer S., Ringelstetter L., Müller C., Zandkarimi F., Merl-Pham J., Bao X., Anastasov N., Kössl J. (2020). GTP Cyclohydrolase 1/Tetrahydrobiopterin Counteract Ferroptosis through Lipid Remodeling. ACS Cent. Sci..

[50] Mao C., Liu X., Zhang Y., Lei G., Yan Y., Lee H., Koppula P., Wu S., Zhuang L., Fang B. (2021). DHODH-mediated ferroptosis defence is a targetable vulnerability in cancer. Nature.

[51] Yang D., Wang J., Li C., Shi L., Zhang M. (2021). Ferroptosis-related gene model to predict overall survival of papillary thyroid carcinoma. Am. J. Otolaryngol..

[52] Anandhan A., Dodson M., Schmidlin C.J., Liu P., Zhang D.D. (2020). Breakdown of an Ironclad Defense System: The Critical Role of NRF2 in Mediating Ferroptosis. Cell Chem. Biol..

[53] Anandhan A., Dodson M., Shakya A., Chen J., Liu P., Wei Y., Tan H., Wang Q., Jiang Z., Yang K. (2023). NRF2 controls iron homeostasis and ferroptosis through HERC2 and VAMP8. Sci. Adv..

[54] Roh J.L., Kim E.H., Jang H., Shin D. (2017). Nrf2 inhibition reverses the resistance of cisplatin-resistant head and neck cancer cells to artesunate-induced ferroptosis. Redox Biol..

[55] Lee J., Roh J.L. (2023). Targeting Nrf2 for ferroptosis-based therapy: Implications for overcoming ferroptosis evasion and therapy resistance in cancer. Biochim. Biophys. Acta Mol. Basis Dis..

[56] Li S., Zhang Y., Zhang J., Yu B., Wang W., Jia B., Chang J., Liu J. (2022). Neferine Exerts Ferroptosis-Inducing Effect and Antitumor Effect on Thyroid Cancer through Nrf2/HO-1/NQO1 Inhibition. J. Oncol..

[57] Kohda A., Kamakura S., Hayase J., Sumimoto H. (2024). The NADPH oxidases DUOX1 and DUOX2 are sorted to the apical plasma membrane in epithelial cells via their respective maturation factors DUOXA1 and DUOXA2. Genes Cell.

[58] Wang B., Yao Z., Wang Z., Yao S., Cen X., Zhang W. (2024). Dysregulated BCL9 Controls Tumorigenicity and Ferroptosis Susceptibility by Binding with Nrf2 in Thyroid Carcinoma. Mol. Carcinog..

[59] Szanto I., Pusztaszeri M., Mavromati M. (2019). H_2_O_2_ Metabolism in Normal Thyroid Cells and in Thyroid Tumorigenesis: Focus on NADPH Oxidases. Antioxidants.

[60] Zhang B.T., Guo M., Yang L.R., Zeng Y., Jiang J. (2025). Mechanistic Aspects of Inflammation and Oxidative Stress and Their Association with Thyroid Cancer Risk. Cancer Med..

[61] Wu J., Chen Z., Duan J., Zhang G. (2025). Mechanisms and pathways of ROS and autophagy in thyroid cancer. Future Sci. OA.

[62] Inoue M., Iizuka Y., Nakamura K., Sato G.E., Mizowaki T. (2023). Role of albumin Cys34 redox state in the progression of differentiated thyroid carcinoma and induction of ferroptosis. Free Radic. Biol. Med..

[63] Rigutto S., Hoste C., Grasberger H., Milenkovic M., Communi D., Dumont J.E., Corvilain B., Miot F., De Deken X. (2009). Activation of dual oxidases Duox1 and Duox2: Differential regulation mediated by camp-dependent protein kinase and protein kinase C-dependent phosphorylation. J. Biol. Chem..

[64] Macvanin M.T., Gluvic Z., Zafirovic S., Gao X., Essack M., Isenovic E.R. (2022). The protective role of nutritional antioxidants against oxidative stress in thyroid disorders. Front. Endocrinol..

[65] Li S., Yuan H., Li L., Li Q., Lin P., Li K. (2025). Oxidative Stress and Reprogramming of Lipid Metabolism in Cancers. Antioxidants.

[66] Cazarin J., Dupuy C., Pires de Carvalho D. (2022). Redox Homeostasis in Thyroid Cancer: Implications in Na(+)/I(-) Symporter (NIS) Regulation. Int. J. Mol. Sci..

[67] Zhao S., Zhao Y., Zhao Y., Wang G. (2023). Pathogenesis and signaling pathways related to iodine-refractory differentiated thyroid cancer. Front. Endocrinol..

[68] Arczewska K.D., Piekiełko-Witkowska A. (2025). The Influence of Micronutrients and Environmental Factors on Thyroid DNA Integrity. Nutrients.

[69] Jin X., Zhu H., Chen X., Yang Y., Song D. (2024). RON receptor tyrosine kinase regulates glycolysis through MAPK/CREB signaling to affect ferroptosis and chemotherapy sensitivity of thyroid cancer cells. Mol. Med. Rep..

[70] Pamarthy D., Behera S.K., Swain S., Yadav S., Suresh S., Jain N., Bhadra M.P. (2023). Diaryl ether derivative inhibits GPX4 expression levels to induce ferroptosis in thyroid cancer cells. Drug Dev. Res..

[71] Wang Z., Zong H., Liu W., Lin W., Sun A., Ding Z., Chen X., Wan X., Liu Y., Hu Z. (2024). Augmented ERO1α upon mTORC1 activation induces ferroptosis resistance and tumor progression via upregulation of SLC7A11. J. Exp. Clin. Cancer Res..

[72] Hammad M., Raftari M., Cesário R., Salma R., Godoy P., Emami S.N., Haghdoost S. (2023). Roles of Oxidative Stress and Nrf2 Signaling in Pathogenic and Non-Pathogenic Cells: A Possible General Mechanism of Resistance to Therapy. Antioxidants.

[73] Gong Z., Xue L., Li H., Fan S., van Hasselt C.A., Li D., Zeng X., Tong M.C.F., Chen G.G. (2024). Targeting Nrf2 to treat thyroid cancer. Biomed. Pharmacother..

[74] Shin D., Kim E.H., Lee J., Roh J.L. (2018). Nrf2 inhibition reverses resistance to GPX4 inhibitor-induced ferroptosis in head and neck cancer. Free Radic. Biol. Med..

[75] Renaud C.O., Ziros P.G., Chartoumpekis D.V., Bongiovanni M., Sykiotis G.P. (2019). Keap1/Nrf2 Signaling: A New Player in Thyroid Pathophysiology and Thyroid Cancer. Front. Endocrinol..

[76] Zimmermann M.B., Köhrle J. (2002). The impact of iron and selenium deficiencies on iodine and thyroid metabolism: Biochemistry and relevance to public health. Thyroid.

[77] Sun L., Zheng G., Zhou M., Zhang Y., Yang Y., Zhang S., Gao L. (2024). In Vitro Ferroptotic and Antitumor Effect of Free or Liposome-Encapsulated Artesunate in Papillary Thyroid Cancer Cells. ACS Omega.

[78] Ling L., Zhang J., Zhang X., Wang P., Ma M., Yin B. (2025). Iodine-131 induces ferroptosis and synergizes with sulfasalazine in differentiated thyroid cancer cells via suppressing SLC7A11. Front. Oncol..

[79] Nikiforov Y.E. (2008). Thyroid carcinoma: Molecular pathways and therapeutic targets. Mod. Pathol..

[80] Li Y., Wang P., Cao J., Liu H. (2026). Multidisciplinary Team Diagnosis and Treatment of well-differentiated thyroid carcinoma: Current Landscape and Future Prospects. Oncologist.

[81] Iravani A., Solomon B., Pattison D.A., Jackson P., Ravi Kumar A., Kong G., Hofman M.S., Akhurst T., Hicks R.J. (2019). Mitogen-Activated Protein Kinase Pathway Inhibition for Redifferentiation of Radioiodine Refractory Differentiated Thyroid Cancer: An Evolving Protocol. Thyroid.

[82] Wang X., Tan X., Zhang J., Wu J., Shi H. (2023). The emerging roles of MAPK-AMPK in ferroptosis regulatory network. Cell Commun. Signal.

[83] Jiang X., Yu M., Wang W.K., Zhu L.Y., Wang X., Jin H.C., Feng L.F. (2024). The regulation and function of Nrf2 signaling in ferroptosis-activated cancer therapy. Acta Pharmacol. Sin..

[84] Fan X., Xie F., Zhang L., Tong C., Zhang Z. (2022). Identification of immune-related ferroptosis prognostic marker and in-depth bioinformatics exploration of multi-omics mechanisms in thyroid cancer. Front. Mol. Biosci..

[85] Kim J.W., Lee J.Y., Oh M., Lee E.W. (2023). An integrated view of lipid metabolism in ferroptosis revisited via lipidomic analysis. Exp. Mol. Med..

[86] Ge M., Niu J., Hu P., Tong A., Dai Y., Xu F., Li F. (2021). A Ferroptosis-Related Signature Robustly Predicts Clinical Outcomes and Associates with Immune Microenvironment for Thyroid Cancer. Front. Med..

[87] Chen W., Fu J., Chen Y., Li Y., Ning L., Huang D., Yan S., Zhang Q. (2021). Circular RNA circKIF4A facilitates the malignant progression and suppresses ferroptosis by sponging miR-1231 and upregulating GPX4 in papillary thyroid cancer. Aging.

[88] Qin Y., Zhang D., Zhang H., Hou L., Wang Z., Yang L., Zhang M., Zhao G., Yao Q., Ling R. (2022). Construction of a ferroptosis-related five-lncRNA signature for predicting prognosis and immune response in thyroid carcinoma. Cancer Cell Int..

[89] Saadh M.J., Bishoyi A.K., Rekha M.M., Verma A., Nanda A., Panigrahi R., Verma R., Gabble B.C. (2025). Dual roles of long non-coding RNAs in thyroid cancer: Regulation of programmed cell death pathways. Med. Oncol..

[90] Xiao W., Lai Y., Yang H., Que H. (2024). Predictive Role of a Novel Ferroptosis-Related lncRNA Pairs Model in the Prognosis of Papillary Thyroid Carcinoma. Biochem. Genet..

[91] Zhao J.Y., Yao J.M., Zhang X.Z., Wang K.L., Jiang S., Guo S.Y., Sheng Q.Q., Liao L., Dong J.J. (2024). A New Ferroptosis-Related Long Non-Coding RNA Risk Model Predicts the Prognosis of Patients with Papillary Thyroid Cancer. World J. Oncol..

[92] Shi J., Wu P., Sheng L., Sun W., Zhang H. (2021). Ferroptosis-related gene signature predicts the prognosis of papillary thyroid carcinoma. Cancer Cell Int..

[93] Luo L., Sun Y., Cao Z. (2025). METTL3-Induced m6A Modification Enhances Hsa_Circ_0136959 Expression to Impair the Tumor Characteristics of Papillary Thyroid Carcinoma via Accelerating Ferroptosis. DNA Cell Biol..

[94] Zhang Z., Zhou D., Qiu X., Xia F., Li X. (2025). N6-methyladenosine-mediated EIF3H promotes anaplastic thyroid cancer progression and ferroptosis resistance by stabilizing β-catenin. Free Radic. Biol. Med..

[95] Ju S.H., Song M., Lim J.Y., Kang Y.E., Yi H.S., Shong M. (2024). Metabolic Reprogramming in Thyroid Cancer. Endocrinol. Metab..

[96] Liao T., Zeng Y., Xu W., Shi X., Shen C., Du Y., Zhang M., Zhang Y., Li L., Ding P. (2025). A spatially resolved transcriptome landscape during thyroid cancer progression. Cell Rep. Med..

[97] Zheng G., Chen S., Ma W., Wang Q., Sun L., Zhang C., Chen G., Zhang S., Chen S. (2025). Spatial and Single-Cell Transcriptomics Unraveled Spatial Evolution of Papillary Thyroid Cancer. Adv. Sci..

[98] Park V.S., Pope L.E., Ingram J.P., Alchemy G.A., Purkal J.J., Murray M.B., Jin S., Andino-Frydman E.Y., Singh S., Chen A. (2025). Lipid Composition Alters Ferroptosis Sensitivity. Cancer Res..

[99] Sokol K.H., Lee C.J., Rogers T.J., Waldhart A., Ellis A.E., Madireddy S., Daniels S.R., House R.R.J., Ye X., Olesnavich M. (2025). Lipid availability influences ferroptosis sensitivity in cancer cells by regulating polyunsaturated fatty acid trafficking. Cell Chem. Biol..

[100] Yu Z., Zhang L., Jiang B., Zhang L., Chen M., Song M. (2026). The ACSL family: Bridging fatty acid metabolism and cell death in cancer progression. Metabolism.

[101] Ping P., Ma Y., Xu X., Li J. (2025). Reprogramming of fatty acid metabolism in thyroid cancer: Potential targets and mechanisms. Chin. J. Cancer Res..

[102] Walther T.C., Farese R.V. (2012). Lipid droplets and cellular lipid metabolism. Annu. Rev. Biochem..

[103] Danielli M., Perne L., Jarc Jovičić E., Petan T. (2023). Lipid droplets and polyunsaturated fatty acid trafficking: Balancing life and death. Front. Cell Dev. Biol..

[104] Safi R., Menéndez P., Pol A. (2024). Lipid droplets provide metabolic flexibility for cancer progression. FEBS Lett..

[105] Li D., Li Y. (2020). The interaction between ferroptosis and lipid metabolism in cancer. Signal Transduct. Target. Ther..

[106] Zhang Y., Du X., Cai S., Cao Y., Zhao D., Xu W., Liao T., Qu N., Shi R., Ji Q. (2025). Developing a thyroid cancer differentiation state classification system using deep residual networks and metabolic signature profiling. NPJ Digit. Med..

[107] Noronha S., Liu Y., Geneti G., Li H., Wu X., Sun D., Gujar V., Furusawa T., Lobanov A., Cam M. (2025). CRISPR-Based Gene Dependency Screens reveal Mechanism of BRAF Inhibitor Resistance in Anaplastic Thyroid Cancer. bioRxiv.

[108] Jomova K., Alomar S.Y., Valko R., Fresser L., Nepovimova E., Kuca K., Valko M. (2026). Interplay of oxidative stress and antioxidant mechanisms in cancer development and progression. Arch. Toxicol..

[109] Lukasiewicz M., Zwara A., Kowalski J., Mika A., Hellmann A. (2024). The Role of Lipid Metabolism Disorders in the Development of Thyroid Cancer. Int. J. Mol. Sci..

[110] Wan Y., Li G., Cui G., Duan S., Chang S. (2025). Reprogramming of Thyroid Cancer Metabolism: From Mechanism to Therapeutic Strategy. Mol. Cancer.

[111] Du J.J., Wang J., Ma K., Ma P. (2026). New insights into the tumor immune microenvironment and immunotherapy of thyroid cancer. Front. Immunol..

[112] Cui K., Wang K., Huang Z. (2024). Ferroptosis and the tumor microenvironment. J. Exp. Clin. Cancer Res..

[113] Zhai X., Lin Y., Zhu L., Wang Y., Zhang J., Liu J., Li L., Lu X. (2024). Ferroptosis in cancer immunity and immunotherapy: Multifaceted interplay and clinical implications. Cytokine Growth Factor Rev..

[114] Tasong J., Sheldon R., Clements A., Abid M.T., Gan A. (2025). Effectiveness of immune checkpoint inhibitor therapy in thyroid cancer: A systematic review. Cancer Treat. Rev..

[115] Chen R., Zou J., Liu J., Kang R., Tang D. (2025). DAMPs in the immunogenicity of cell death. Mol. Cell.

[116] Huang Y., Xie Z., Li X., Chen W., He Y., Wu S., Li X., Hou B., Sun J., Wang S. (2021). Development and validation of a ferroptosis-related prognostic model for the prediction of progression-free survival and immune microenvironment in patients with papillary thyroid carcinoma. Int. Immunopharmacol..

[117] Zhu L., Li X.J., Gangadaran P., Jing X., Ahn B.C. (2023). Tumor-associated macrophages as a potential therapeutic target in thyroid cancers. Cancer Immunol. Immunother..

[118] Yang Y., Wang Y., Guo L., Gao W., Tang T.L., Yan M. (2022). Interaction between macrophages and ferroptosis. Cell Death Dis..

[119] Tang D., Kroemer G., Kang R. (2024). Ferroptosis in immunostimulation and immunosuppression. Immunol. Rev..

[120] Li Z., Ma J., Guan H., Lai J., Xu F., Cao G. (2025). FTO-mediated the destabilization of RASGRF1 mRNA impedes thyroid cancer progression and suppresses macrophage M2 polarization. Cell Biol. Toxicol..

[121] Garza B., Calhoun J., Norman P., Cline N., Kichula K.M., Farias T.D.J., Sams S., Boorgula M.P., Danhorn T., Haugen B.R. (2025). Tumor-infiltrating T Lymphocytes Recognize Thyroid-specific and Neo-antigens in Follicular Cell-derived Thyroid Cancers. J. Clin. Endocrinol. Metab..

[122] Lin R., Fogarty C.E., Ma B., Li H., Ni G., Liu X., Yuan J., Wang T. (2021). Identification of ferroptosis genes in immune infiltration and prognosis in thyroid papillary carcinoma using network analysis. BMC Genom..

[123] Ebrahimnezhad M., Valizadeh A., Yousefi B. (2025). Ferroptosis and immunotherapy: Breaking barriers in cancer treatment resistance. Crit. Rev. Oncol. Hematol..

[124] Rumiano L., Manzo T. (2025). Lipids guide T cell antitumor immunity by shaping their metabolic and functional fitness. Trends Endocrinol. Metab..

[125] Ma J., Li Z., Xu J., Lai J., Zhao J., Ma L., Sun X. (2024). PRDM1 promotes the ferroptosis and immune escape of thyroid cancer by regulating USP15-mediated SELENBP1 deubiquitination. J. Endocrinol. Investig..

[126] Zhang F., Ma Y., Li D., Wei J., Chen K., Zhang E., Liu G., Chu X., Liu X., Liu W. (2024). Cancer associated fibroblasts and metabolic reprogramming: Unraveling the intricate crosstalk in tumor evolution. J. Hematol. Oncol..

[127] Chen Z., Han F., Du Y., Shi H., Zhou W. (2023). Hypoxic microenvironment in cancer: Molecular mechanisms and therapeutic interventions. Signal Transduct. Target. Ther..

[128] Ferrari S.M., Fallahi P., Galdiero M.R., Ruffilli I., Elia G., Ragusa F., Paparo S.R., Patrizio A., Mazzi V., Varricchi G. (2019). Immune and Inflammatory Cells in Thyroid Cancer Microenvironment. Int. J. Mol. Sci..

[129] Zhang L., Xu S., Cheng X., Wu J., Wang Y., Gao W., Bao J., Yu H. (2023). Inflammatory tumor microenvironment of thyroid cancer promotes cellular dedifferentiation and silencing of iodide-handling genes expression. Pathol. Res. Pract..

[130] Chen Y., Fang Z.M., Yi X., Wei X., Jiang D.S. (2023). The interaction between ferroptosis and inflammatory signaling pathways. Cell Death Dis..

[131] Dang Q., Sun Z., Wang Y., Wang L., Liu Z., Han X. (2022). Ferroptosis: A double-edged sword mediating immune tolerance of cancer. Cell Death Dis..

[132] Leboulleux S., Boucai L., Busaidy N., Durante C., Fagin J.A., Fazeli S., Gianoukakis A.G., Haugen B.R., Kang H., Konda B. (2025). Redifferentiation therapy in unresectable or metastatic radioactive iodine refractory thyroid cancer: An International Thyroid Oncology Group statement. Lancet Diabetes Endocrinol..

[133] Kim M., Jin M., Jeon M.J., Kim E.Y., Shin D.Y., Lim D.J., Kim B.H., Kang H.C., Kim W.B., Shong Y.K. (2023). Lenvatinib Compared with Sorafenib as a First-Line Treatment for Radioactive Iodine-Refractory, Progressive, Differentiated Thyroid Carcinoma: Real-World Outcomes in a Multicenter Retrospective Cohort Study. Thyroid.

[134] Valerio L., Matrone A. (2025). Multikinase and highly selective kinase inhibitors in the neoadjuvant treatment of patients with thyroid cancer. Explor. Target. Antitumor Ther..

[135] Zheng J., Sato M., Mishima E., Sato H., Proneth B., Conrad M. (2021). Sorafenib fails to trigger ferroptosis across a wide range of cancer cell lines. Cell Death Dis..

[136] Chen X., Chen X., Xie W., Ge H., He H., Zhang A., Zheng J. (2025). BRAF-activated ARSI suppressed EREG-mediated ferroptosis to promote BRAF(V600E) (mutant) papillary thyroid carcinoma progression and sorafenib resistance. Int. J. Biol. Sci..

[137] Gong Z., Xue L., Wei M., Liu Z., Vlantis A.C., van Hasselt C.A., Chan J.Y.K., Li D., Zeng X., Tong M.C.F. (2021). The Knockdown of Nrf2 Suppressed Tumor Growth and Increased the Sensitivity to Lenvatinib in Anaplastic Thyroid Cancer. Oxid. Med. Cell Longev..

[138] Xu S., Liang Q., Li H., Zhou H., Xu Z., Yan Y., Zhang Y., Ye R., You X. (2025). Combination of astragalus polysaccharide with Diosbulbin B exerts an enhanced antitumor effect in BRAF(mut) papillary thyroid cancer with decreased liver toxicity. Cancer Cell Int..

[139] Yang X., Zhang M., Xia W., Mai Z., Ye Y., Zhao B., Song Y. (2023). CHAC1 promotes cell ferroptosis and enhances radiation sensitivity in thyroid carcinoma. Neoplasma.

[140] Yang F., Yu W., Yu Q., Liu X., Liu C., Lu C., Liao X., Liu Y., Peng N. (2023). Mitochondria-Targeted Nanosystem with Reactive Oxygen Species-Controlled Release of CO to Enhance Photodynamic Therapy of PCN-224 by Sensitizing Ferroptosis. Small.

[141] Wang K., Zhang Y., Xing Y., Wang H., He M., Guo R. (2024). Current and future of immunotherapy for thyroid cancer based on bibliometrics and clinical trials. Discov. Oncol..

[142] Wang W., Li H., Liang S., Hu Y., Ding J., Wu X., Hua D. (2025). Bridging the gap: Ferroptosis of immune cells in the tumor microenvironment. Front. Immunol..

[143] Liang L., Chen Z., Lei D., Mo C., Lan D., Ke J., Wang W., Yang Z., Guo X., Chen D. (2025). APG-115 Induces SLC7A11-Mediated Ferroptosis and Upregulates PD-L1 Expression in Thyroid Cancer. ACS Omega.

[144] Xiong D., Li Z., Zuo L., Ge J., Gu Y., Zhang E., Zhou X., Yu G., Sang M. (2024). Comprehensive Analysis Reveals That ISCA1 Is Correlated with Ferroptosis-Related Genes Across Cancers and Is a Biomarker in Thyroid Carcinoma. Genes.

[145] Wang H.H., Ma J.N., Zhan X.R. (2021). Circular RNA Circ_0067934 Attenuates Ferroptosis of Thyroid Cancer Cells by miR-545-3p/SLC7A11 Signaling. Front. Endocrinol..

[146] Sun S., Shen J., Jiang J., Wang F., Min J. (2023). Targeting ferroptosis opens new avenues for the development of novel therapeutics. Signal Transduct. Target. Ther..

